# Fgk3 glycogen synthase kinase is important for development, pathogenesis, and stress responses in *Fusarium graminearum*

**DOI:** 10.1038/srep08504

**Published:** 2015-02-23

**Authors:** Jun Qin, Guanghui Wang, Cong Jiang, Jin-Rong Xu, Chenfang Wang

**Affiliations:** 1 State Key Laboratory of Crop Stress Biology for Arid Areas, College of Plant Protection, Northwest A&F University, Yangling, Shaanxi, China; 2 Department of Botany and Plant Pathology, Purdue University, USA

## Abstract

Wheat scab caused by *Fusarium graminearum* is an important disease. In a previous study, the *FGK3* glycogen synthase kinase gene orthologous to mammalian GSK3 was identified as an important virulence factor. Although GSK3 orthologs are well-conserved, none of them have been functionally characterized in fungal pathogens. In this study, we further characterized the roles of *FGK3* gene. The Δ*fgk3* mutant had pleiotropic defects in growth rate, conidium morphology, germination, and perithecium formation. It was non-pathogenic in infection assays and blocked in DON production. Glycogen accumulation was increased in the Δ*fgk3* mutant, confirming the inhibitory role of Fgk3 on glycogen synthase. In *FGK3*-GFP transformants, GFP signals mainly localized to the cytoplasm in conidia but to the cytoplasm and nucleus in hyphae. Moreover, the expression level of *FGK3* increased in response to cold, H_2_O_2_, and SDS stresses. In the Δ*fgk3* mutant, cold, heat, and salt stresses failed to induce the expression of the stress response-related genes *FgGRE2, FgGPD1, FgCTT1*, and *FgMSN2*. In the presence of 80 mM LiCl, a GSK3 kinase inhibitor, the wild type displayed similar defects to the Δ*fgk3* mutant. Overall, our results indicate that *FGK3* is important for growth, conidiogenesis, DON production, pathogenicity, and stress responses in *F*. *graminearum*.

F*usarium graminearum* is the predominant species that causes Fusarium head blight (WHB) or scab of wheat and barley worldwide^1^,^2^. Under favorable conditions, it can cause severe yield losses, and often contaminates infected grains with harmful mycotoxins. One of the mycotoxins produced by *F. graminearum* is trichothecene mycotoxin deoxynivalenol (DON), which is a potent inhibitor of eukaryotic protein synthesis and an important virulence factor^3^. The trichothecene biosynthetic gene clusters and biosynthesis pathways in *F. graminearum* and related species have been extensively studied in the past decade^4^,^5^.

To better understand fungal pathogenesis, we systematically characterized protein kinase genes in a previous study^6^. In total, 42 of them were found to be important for pathogenicity or virulence in infection assays with flowering wheat heads and corn stalks. In addition to components of the well-conserved cAMP signaling and MAP kinase pathways, we found 31 protein kinase genes that had not been previously characterized as important pathogenicity factors. One of them, FGSG_07329, is orthologous to the mammalian GSK3β glycogen synthase kinase gene^7^.

GSK3 orthologs from different eukaryotic organisms, including mammals, insects, fungi, nematodes, and protozoa, have similar structures and well-conserved ATP binding sites^8^. Although GSK3 was first characterized as a Ser/Thr protein kinase responsible for the phosphorylation and inactivation of glycogen synthase, later studies have shown that GSK3 functions in multiple cellular processes^7^. In addition to enzymes involved in metabolism, its substrates include structural and signaling proteins, as well as transcription factors. In mammalian cells, GSK3 plays roles in many signaling pathways that are involved in cell proliferation and differentiation, microtubule dynamics, development, and oncogenesis^9^,^10^. Alzheimer's disease is one of several human diseases that are known to be related to the hyperactivity of GSK3^11^. In plants, GSK3 kinases function in hormonal signaling networks that involve brassinosteroids, abscisic acid, and auxin during growth and development^12^. They also play roles in floral organ development and cell expansion, as well as in responses to biotic and abiotic stresses^13^.

Although *F. graminearum* contains only one, the budding yeast *Saccharomyces cerevisiae* has four *GSK3* orthologs, *MCK1*, *RIM11*, *MRK1*, and *YGK3*^14^. Whereas *RIM11* is paralogous to *MRK1*, *MCK1* is paralogous to *YGK3*. Both pairs arose from the genome duplication event. *MCK1* plays roles in mitotic chromosome segregation and regulation of Ime1, which interacts with Ume6 to activate the transcription of early meiosis genes^15^. *MCK1* also facilitates cell cycle delay in response to calcium stress^16^. *RIM11* is also required for entering meiosis by phosphorylation of Ime1 and Ume6^17^. The expression of *RIM11* increases in response to DNA replication stress^18^. In contrast, both *YGK3* and *MRK1* play roles in protein degradation and Msn2-dependent transcription^14^,^19^. *YGK3* is also required for optimal growth under zinc-limiting conditions but the *MRK1* deletion mutant had no obvious defects in growth, colony morphology, and glycogen accumulation^20^.

In *Cryptococcus neoformans*, *GSK3* is involved in the sterol regulatory element-binding protein (SREBP) pathway, which plays a role in the regulation of cholesterol and lipid metabolism in mammalian cells^21^,^22^. However, in *C. neoformans*, the SREBP pathway also plays roles in the connection between oxygen sensing, CoCl_2_ sensitivity, and virulence^21^. The X-ray structure of *Ustilago maydis* Gsk3 was analyzed, and type- II kinase inhibitors were demonstrated to have inhibitory effects on Gsk3 and the potential to be developed into anti-fungal agents^23^. In the fission yeast *Schizosaccharomyces pombe*, the *skp1* gene encodes a protein that shares 67% amino acid sequence identity with mammalian GSK3β. The *skp1* deletion mutant was sensitive to heat shock and exhibited defects in sporulation. Overexpression of *skp1* complemented the defects of the *cdc14* mutant in cytokinesis^24^.

GSK3 orthologs are well conserved in filamentous ascomycetes. However, none of them have been functionally characterized. All of the plant pathogenic fungi that have been sequenced contain at least one GSK3 ortholog, but their functions in plant infection have not been identified. In this study, we characterized the roles of the GSK3 ortholog in *F. graminearum* (named *FGK3* for *F. graminearum* *GSK3*). Compared to the wild type strain, the Δ*fgk3* mutant had pleiotropic defects in growth rate, conidium morphology, germination, perithecium formation, and glycogen accumulation. Moreover, the Δ*fgk3* mutant was non-pathogenic in infection assays with flowering wheat heads and blocked in DON production. The expression level of *FGK3* increased in responses to different environmental stressors. In the Δ*fgk3* mutant, the up-regulation of the *FgGRE2*, *FgGPD1*, *FgCTT1*, and *FgMSN2* genes that are known to be involved in general stress responses by environmental stresses was diminished. These results indicate that the *FGK3* gene is important for hyphal growth, conidiogenesis, DON production, pathogenicity, glycogen accumulation, and stress responses in *F*. *graminearum*.

## Results

### FGSG_07329 (*FGK3*) encodes a typical GSK3 protein kinase

*FGK3* encodes a 394 amino acid protein that contains a well-conserved protein kinase domain from residues 35 to 318. Like other GSK3 orthologs, the rest of Fgk3 (apart from the kinase domain) is not similar to any known motif or domain. Fgk3 shares 65% identity in amino acid sequences with the human GSK3β kinase. Its orthologs are also well conserved in filamentous fungi (Fig. S1). Phylogenetic analysis showed that FOPG_04806 of *Fusarium oxysporum* and FVEG_07936 of *F. verticillioides* are closely related to *FGK3*, both sharing 99% sequence identity.

### The Δ*fgk3* mutant is defective in growth, conidiogenesis, and conidium germination

**Figure 1:**
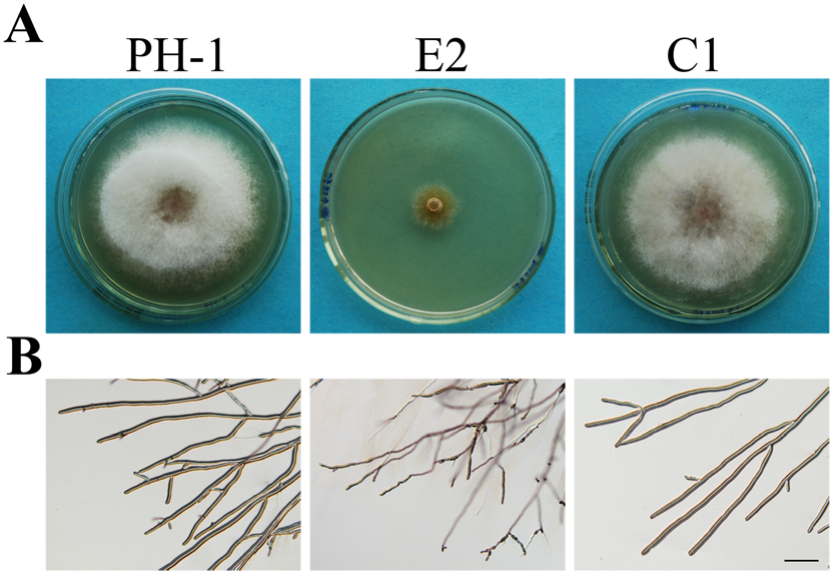
Colony morphology and growth defects of the Δ*fgk3* mutant. (A). Three-day-old CM cultures of the wild type PH-1, the Δ*fgk3* mutant E2, and the complemented transformant C1. (B). Hyphal growth at the edge of PH-1, E2, and C1 colonies. Bar = 200 μm.

The *FGK3* gene replacement construct was generated and transformed into the wild-type strain PH-1 in a previous study^6^. The resulting Δ*fgk3* transformants were confirmed by PCR and Southern blot analysis (Fig. S2). In comparison with PH-1, the Δ*fgk3* mutant E2 was significantly reduced in growth rate on CM plates (Fig. 1A; Table S1). Colonies formed by mutant E2 had yellowish pigmentation and limited aerial hyphal growth (Fig. 1A). Microscopic examination further revealed that the Δ*fgk3* mutant had uneven hyphal width (Fig. 1B).

**Figure 2:**
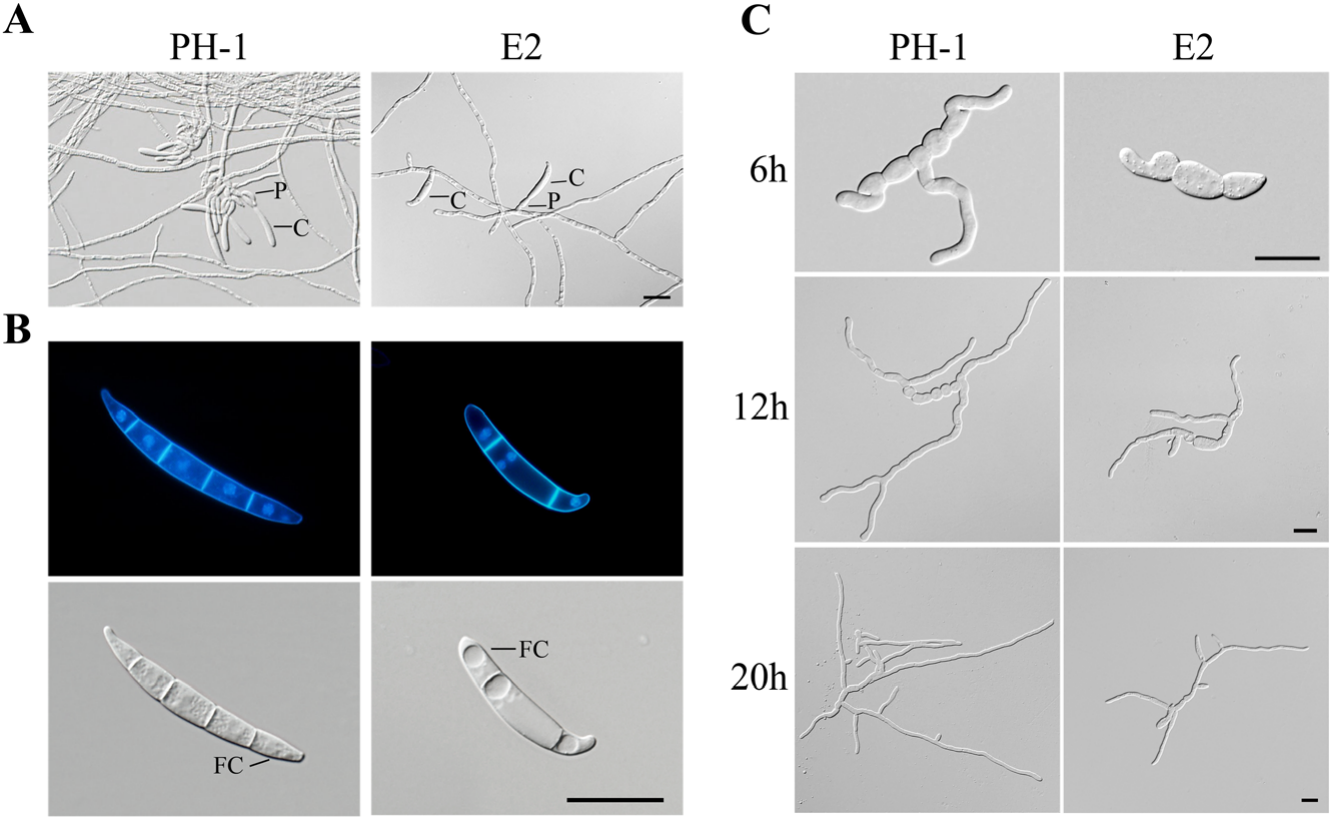
Defects of the Δ*fgk3* mutant in conidiogenesis, conidium morphology, and germination. (A). CMC cultures of the wild type PH-1 and Δ*fgk3* mutant E2 were examined for conidiophores and phialides. P, phialide; C, conidium. (B). Conidia of PH-1 and E2 were stained with DAPI and Calcofluor white and examined by DIC or epifluorescence microscopy. FC, foot cell. (C). Conidia of PH-1 and E2 were incubated in liquid YEPD for 6, 12, and 20 h and examined for germination and germ tube growth. Bar = 20 μm for all the panels.

The Δ*fgk3* mutant was also significantly reduced in conidiation (Table S1). Microscopic examination showed that deletion of *FGK3* resulted in defects in phialide formation. Conidia were often directly formed on hyphal branches instead of on phialides, which may be directly responsible for reduced conidiation in the Δ*fgk3* mutant (Fig. 2A). The Δ*fgk3* mutant was also defective in conidium morphology. Conidia of the Δ*fgk3* mutant were shorter and had fewer septa (Fig. 2B). Additionally, the tip compartments of mutant conidia were often elongated and strongly curved, forming hook-like structures (Fig. 2A; 2B), while the middle compartments of conidia often contained multiple nuclei (Fig. 2B). These results indicate that *FGK3* is important for conidiogenesis and septation or cytokinesis during conidium formation.

When incubated in liquid YEPD, conidia of the Δ*fgk3* mutant were able to germinate from the end and middle compartments like the wild type (Fig. 2C). However, germ tube growth appeared to be reduced in mutant E2. After incubation for 6 h, germ tubes of mutant E2 were shorter than those of PH-1 (Fig. 2C). By 20 h, germ tubes of PH-1 also had branched more than those of the Δ*fgk3* mutant (Fig. 2C). The defect of the Δ*fgk3* mutant in germ tube growth was consistent with its reduced growth rate.

### The Δ*fgk3* mutant is blocked in sexual reproduction

**Figure 3:**
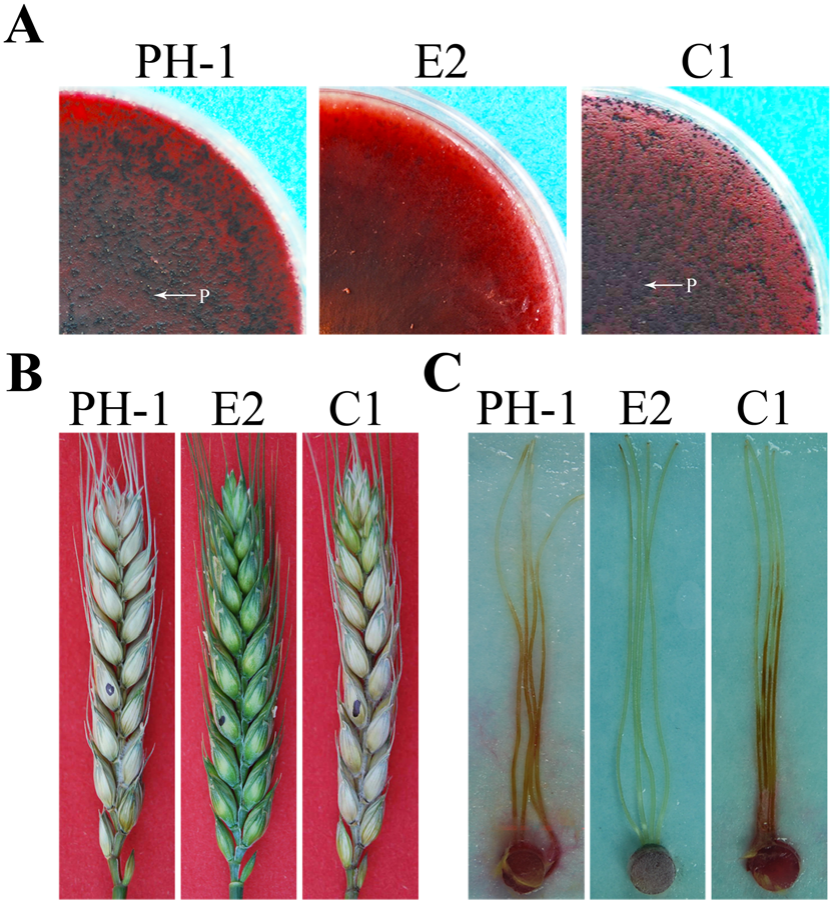
Assays for defects of the Δ*fgk3* mutant in plant infection and sexual reproduction. (A). Self-crossing plates of the wild type PH-1, Δ*fgk3* mutant E2, and complemented strain C1 at 14 days post-fertilization (dpf). Arrows point to perithecia produced by PH-1 and C1. Mutant E2 failed to produce perithecia. (B). Flowering wheat heads were inoculated with conidia of PH-1, E2, and C1, and photographed at 14 days post-inoculation (dpi). (C). Corn silks were inoculated with culture blocks and examined at 6 dpi.

Because ascospores play a critical role in the infection cycle of *F. graminearum*, we also assayed sexual reproduction with the Δ*fgk3* mutant on carrot agar plates as described^25^. At 14 days post-fertilization (dpf), the wild type produced mature perithecia with ascospore cirrhi (Fig. 3A). Under the same conditions, the Δ*fgk3* mutant failed to produce perithecia and protoperithecia (Fig. 3A), suggesting that *FGK3* plays an essential role in sexual reproduction in *F. graminearum*. The Δ*fgk3* mutant must be blocked in the early sexual developmental processes and have lost female fertility.

### *FGK3* is important for plant infection and DON production

In infection assays with flowering wheat heads, the Δ*fgk3* mutant rarely caused symptoms on the inoculated wheat kernels and never spread to neighboring spikelets on the same head (Fig. 3B). The average disease index of the mutant was less than 0.5 (Table 1), which was significantly lower than that of PH-1. In infection assays with corn silks, the Δ*fgk3* mutant also was defective in plant infection. PH-1 caused extensive lesions spreading along corn silks 5 dpi, but the Δ*fgk3* mutant did not cause any discoloration beyond the inoculation sites (Fig. 3C). These results indicated that the Δ*fgk3* mutant was significantly reduced in virulence. Therefore, *FGK3* must play a critical role in plant infection and spreading in *F. graminearum*.

Because DON is an important virulence factor in *F. graminearum*^26^,^27^, we then assayed DON production in diseased wheat kernels. Whereas DON content in PH-1 exceeded 1,600 ppm, DON production in the Δ*fgk3* mutant was not detectable in inoculated wheat kernels (Table 1). Therefore, in addition to its reduced growth rate, defects of the Δ*fgk3* mutant in DON biosynthesis may also contribute to its defects in plant infection.

### Complementation assays and subcellular localization of *FGK3*-GFP fusion proteins

The *FGK3*-GFP fusion construct was generated by the yeast gap repair approach^28^ and transformed into the Δ*fgk3* mutant. The resulting Δ*fgk3*/*FGK3*-GFP transformant C1 was identified by PCR and confirmed by Southern blot analysis. It was normal in growth (Fig. 1), sexual reproduction, and plant infection (Fig. 3). Thus, fusion with GFP had no effect on *FGK3* function, and expression of *FGK3*-GFP rescued the defects of the Δ*fgk3* mutant.

**Figure 4:**
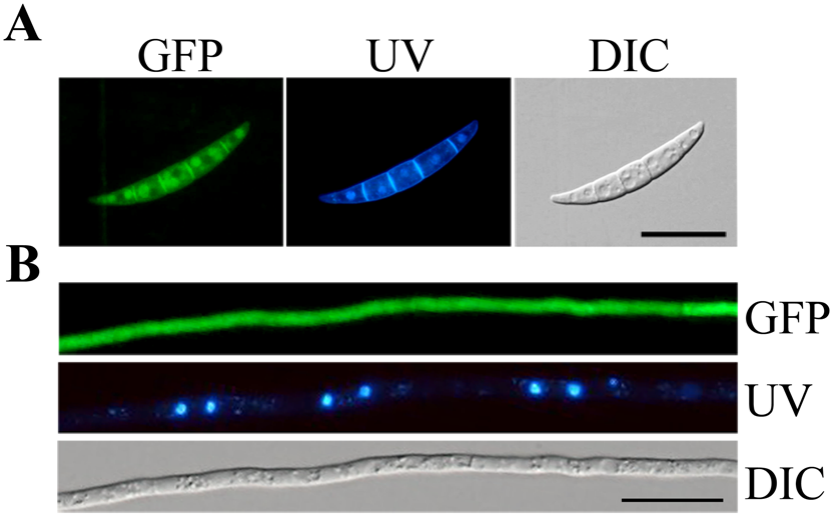
Subcellular localization of Fgk3-GFP fusion proteins. Conidia (A) and 12 h germlings (B) of the Δ*fgk3/FGK3*-GFP transformant (C1) were stained with DAPI and Calcofluor white before being examined by DIC and epifluorescence microscopy (GFP or UV). GFP signals were present in the cytoplasm in conidia and in both the cytosol and nuclei in 12 h germlings. Bar = 20 μm for all the panels.

Interestingly, when examined for GFP signal in the Δ*fgk3*/*GSK3*-GFP transformant C1, Fgk3-GFP mainly localized to the cytoplasm in conidia (Fig. 4A). However, GFP signals were observed in both the cytoplasm and nucleus in 12 h germlings or hyphae (Fig. 4B). The alteration in the subcellular localization of Fgk3-GFP fusion may be related to its diverse functions in different stages.

### Deletion of *FGK3* increased glycogen accumulation in conidia

**Figure 5:**
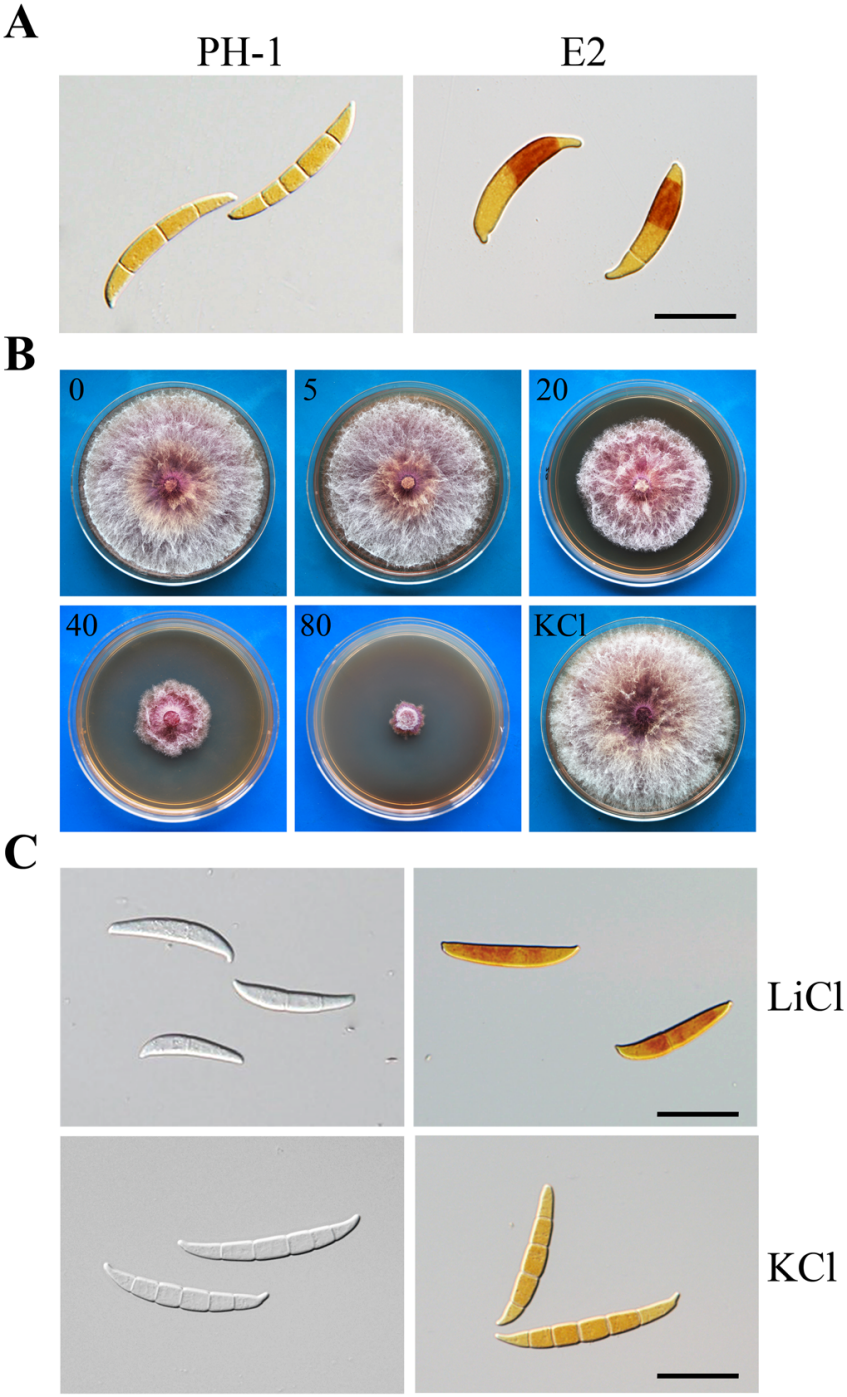
Assays for glycogen accumulation and inhibitory effects of lithium chloride. (A). Conidia of the wild type PH-1 and Δ*fgk3* mutant E2 were stained for glycogens (brown staining) with 60 mg/ml KI and 10 mg/ml of I_2_. (B). PDA cultures of PH-1 were incubated for 5 days in the presence of 5, 20, 40, and 80 mM LiCl. Cultures with 80 mM KCl were included as the control. (C). Conidia of PH-1 produced in CMC cultures with 80 mM LiCl or KCl were stained for glycogens. Bar = 20 μm for all the panels.

In mammalian cells, GSK3 is a glycogen synthase kinase that inhibits the activity of glycogen synthase. To determine the effect of *FGK3* deletion on glycogen synthesis, we stained glycogen in *F. graminearum* conidia as described^29^. In comparison with the wild-type conidia, mutant conidia accumulated more glycogen, although they were defective in morphology (Fig. 5A). However, the distribution of glycogen appeared to be uneven among different conidium compartments in the Δ*fgk3* mutant (Fig. 5A). These results indicated that deletion of *FGK3* increases glycogen synthase activities in the Δ*fgk3* mutant.

### Lithium chloride treatment mimics deletion of *FGK3*

Lithium is known to be an inhibitor of mammalian GSK3β^30^. When treated with increasing concentrations of LiCl, from 5 to 80 mM, the growth rate of PH-1 on PDA was reduced accordingly in increments. In the presence of 80 mM LiCl, hyphal growth in PH-1 was reduced to a level comparable with that of the Δ*fgk3* mutant (Fig. 5B; Table S1). Colonies of cultures treated with 80 mM LiCl were more compact, and had darker pigmentation and fewer aerial hyphae than those of untreated PH-1 cultures. Their colony morphology was similar to that of the Δ*fgk3* mutant. Under the same conditions, PH-1 cultures had no obvious changes in growth rate or colony morphology in the presence of up to 80 mM KCl (Fig. 5B).

Conidia produced by PH-1 in CMC with 80 mM LiCl also had morphological defects (Fig. 5C), although LiCl treatment did not impact conidiation (Table S1). In the presence of 80 mM LiCl, PH-1 produced smaller conidia with curved tip compartments and fewer septa, akin to the Δ*fgk3* mutant. These conidia also accumulated a higher degree of glycogen (Fig. 5C). In contrast, conidia produced by PH-1 in CMC cultures with 80 mM KCl had normal morphology and glycogen accumulation (Fig. 5C). Together, these results indicate that lithium treatment inhibits Fgk3 activities and most of its functions.

### Microtubule bundling at the septal pores is abnormal in the Δ*fgk3* mutant

Severe defects of conidium morphology in the Δ*fgk3* mutant may be due to abnormal microtubule organization, which is normally a key component of the cytoskeleton involved in maintaining cellular structures. To test this hypothesis, we introduced the *TUB1*-GFP construct^31^ into PH-1 and the Δ*fgk3* mutant. In *TUB1*-GFP transformants of PH-1 (T1-P10), microtubule filaments extended from one end of the conidia to the other, and microtubule bundling was visible at the septal pore (Fig. S3). In *TUB1*-GFP transformants of the Δ*fgk3* mutant (Δ*fgk3*-tub), microtubule filaments were also visible in conidia (Fig. S3). No significant difference was observed between the organization and intensities of microtubule filaments between the wild type and mutant transformants. However, microtubule bundling at the septal pore appeared to be loose in the mutant conidia, suggesting that the mutant may have abnormal septal pores. Deletion of *FGK3* may affect the completion of septum formation in *F. graminearum*.

### *FGK3* is involved in responses to various stresses

Because GSK3 orthologs are important for responses to heat and salt stresses in *S. cerevisiae*^32^,^33^ and transgenic *Arabidopsis* plants expressing the wheat *TaGSK1* has increased salt tolerance^34^, we assayed the sensitivity the Δ*fgk3* mutant to different stresses. When the wild-type strain PH-1 was cultured on CM plates with 0.7 M NaCl, 0.05% H_2_O_2_, 0.01% SDS, or 0.3 M Congo Red, the growth rate was reduced in comparison with regular CM plates (Fig. S4). However, the Δ*fgk3* mutant almost had no detectable growth in the presence of any of these chemicals in the medium (Fig. S4). Because the mutant was severely restricted in growth, it is difficult to conclude whether the Δ*fgk3* mutant had increased sensitivities to any of these stresses.

To further determine the role of *FGK3* in stress responses, we assayed its expression in PH-1 cultures grown under various stress conditions. Germlings grown in 16 h YEPD cultures were harvested and treated with 0.7 M NaCl, 0.05% H_2_O_2_, or 0.01% SDS for 1 h at 25°C or incubated at 4°C and 37°C for 1 h. RNA samples were then isolated from hyphae collected from these cultures. When assayed by qRT-PCR, the expression level of *FGK3* was significantly (>2-fold) increased in response to cold, H_2_O_2_, and SDS stresses (Fig. S5A). However, *FGK3* expression was increased only an approximate 1.5-fold when cultured under heat and salt stress conditions.

In addition to liquid cultures, we also assayed the expression of *FGK3* in aerial hypha of PH-1 grown on PDA plates in response to cold and heat shock. The expression level of *FGK3* increased to 7- and 53-fold after incubation at 4°C (cold treatment) for 4 h and 20 h, respectively (Fig. S5B). In contrast, when cultured at 37°C (heat treatment), the expression of *FGK3* increased to only 1.5-fold and 2.2-fold at 4 h and 20 h, respectively (Fig. S5B). Therefore, cold, heat, oxidative, and SDS stresses increased the expression of *FGK3* to different levels in either aerial hypha or hypha in liquid cultures. However, 1 h-treatment of salt stress had little or no effect on the expression of *FGK3*.

### Deletion of *FGK3* affects the expression of selected stress response genes

To further determine the role of *FGK3* in stress responses, we assayed the expression of the *F. graminearum* homologs of yeast *GRE2*, *GPD1*, and *CTT1* genes in the Δ*fgk3* mutant. In yeast, *GRE2* is a NADPH-dependent methylglyoxal reductase whose expression is induced by general stresses including osmotic, ionic, oxidative, heavy metal, and heat stresses^35^. *GPD1* encodes a NAD-dependent glycerol-3-phosphate dehydrogenase that is essential for growth under osmotic stress^36^. *CTT1* encodes the cytosolic catalase, which is vital for survival under severe osmotic stress and heat shock^37^.

**Figure 6:**
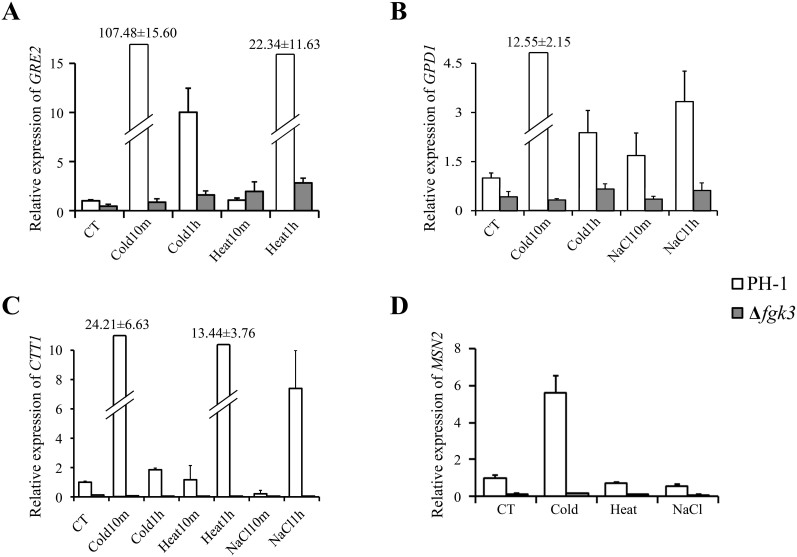
The expression levels of *FgGPD1*, *FgGRE2*, *FgCTT1*, and *FgMSN2* assayed by qRT-PCR. After incubation for 16 h at 25°C, liquid YEPD cultures of PH-1 and the Δ*fgk3* mutant were further incubated at 4°C or 37°C or in the presence of 0.7 M NaCl for 10 min and 1 h, respectively. RNA samples were then isolated from germlings harvested by filtration and used in qRT-PCR assays for the expression levels of (A) *FgGPD1*, (B) *FgGRE2,* (C) *FgCTT1*, and (D) *FgMSN2*. CT: control, untreated PH-1. Mean and standard deviations were calculated with results from three independent replicates.

The expression of *FgGRE2* in PH-1 was up-regulated over 100-fold and 22-fold after incubation for 10 min at 4°C and 1 h at 37°C, respectively (Fig. 6A). However, under the same conditions, *FgGRE2* expression increased less than 2-fold and 7-fold in the Δ*fgk3* mutant. Expression of *FgGPD1* in PH-1 increased 12-fold after incubation for 10 min at 4°C and 3-fold after 1 h in 0.7 M NaCl, but remained relatively unchanged in the mutant (Fig. 6B). The expression of *FgCTT1* in the wild type increased between 7- to 24-fold in response to cold, heat, and salt stresses at different points in time. However, its expression was not detected or barely detectable in the Δ*fgk3* mutant in cultures grown under various stress conditions (Fig. 6C). Furthermore, deletion of *FGK3* reduced the expression of *FgCTT1* to one eighth under normal culture conditions, indicating that *FGK3* is important for *FgCTT1* expression. Therefore, deletion of *FGK3* blocked the up-regulation of *FgGRE2*, *FgGPD1*, and *FgCTT1* by cold, heat, or salt stresses. *FGK3* must play an important role in responses to different environmental stresses in *F. graminearum*.

### Fgk3 physically interacts with FgMsn2

**Figure 7:**
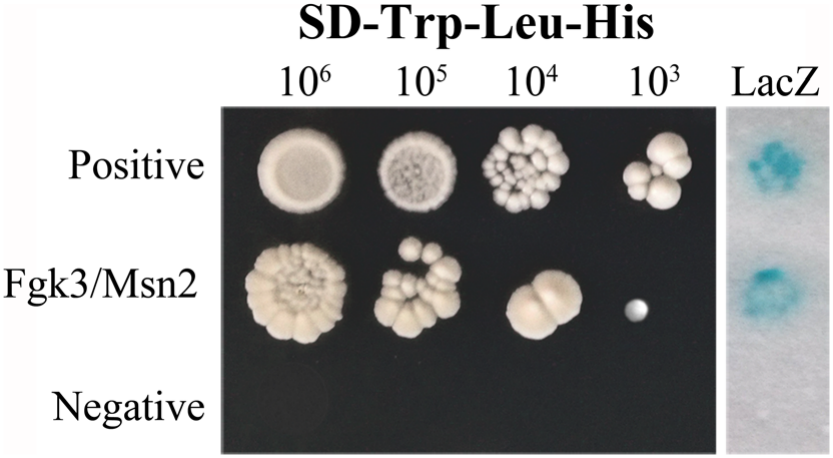
Yeast two hybrid assays to detect the interaction of Fgk3 with FgMsn2. (A). Different cell concentrations (cells/ml) of the yeast transformants expressing the labeled bait and prey constructs were assayed for growth on SD-Leu-Trp-His plates. Positive and negative controls were provided in the BD Matchmaker library construct kit. (B). The same set of yeast transformants was assayed for β-galactosidase activities.

When the promoter sequences of the *FgGRE2*, *FgGPD1*, and *FgCTT1* genes were analyzed, we found that all of them contain a stress response element (STRE, CCCCT, or AGGGG)^35^. In the budding yeast, GSK3 kinases are essential for the STRE-binding activity of Msn2^19^, a transcriptional activator that induces gene expression. To determine whether Fgk3 directly interacts with *FgMSN2* (FGSG_06871) of *F*. *graminearum*, we assayed their interactions by yeast two-hybrid assays. The Fgk3 bait construct and FgMsn2 prey construct were co-transformed into yeast strain AH109. The resultant Trp+ Leu+ transformants were able to grow on the SD-Trp-Leu-His plate and had LacZ activities (Fig. 7). These results suggest that Fgk3 directly interacts with FgMsn2, which may also function as a transcriptional regulator of stress responsive genes in *F. graminearum*.

### Stress-induced expression of *FgMSN2* requires the Fgk3 kinase

In *S. cerevisiae*, mutants deleted of all four GSK3 homologs are defective in the transcription of Msn2-dependend stress responsive genes. Therefore, we assayed the expression of *FgMSN2* in the Δ*fgk3* mutant by qRT-PCR. RNA samples were isolated from hyphae harvested from 16 h YEPD cultures and further incubated at 4°C or 37°C or in the presence of 0.7 M NaCl for 10 min. In response to cold treatment, the expression level of *FgMSN2* increased 5.6-fold in PH-1, but only 1.3-fold in the Δ*fgk3* mutant. In response to heat and salt stresses, there were no significant changes in *FgMSN2* expression in either PH-1 or the Δ*fgk3* mutant (Fig. 6D). These results showed that Fgk3 not only interacts with FgMsn2, but is also involved in the up-regulation of *FgMSN2* expression in response to cold temperatures.

## Discussion

The *FGK3* gene encodes a typical glycogen synthase kinase with a highly conserved protein kinase domain. In animals and plants, GSK3 kinases are known to be related to growth and differentiation^38^,^39^. The budding yeast *S. cerevisiae* has four GSK3 homologs, *MCK1*, *RIM11*, *MRK1*, and *YGK3*. Whereas *MCK1* is required for growth at extreme temperatures, the other three have no significant effect on vegetative growth^14^,^40^ although overexpression of *RIM11* increases filamentous growth in *S. cerevisiae*^41^. However, in *F. graminearum*, the Δ*fgk3* mutant had severe defects in vegetative growth, with a 90% reduction in growth rate. These results indicate that *FGK3* may have a more pronounced effect on vegetative growth in filamentous fungi than in yeasts.

In comparison with the wild type, the Δ*fgk3* mutant also was reduced approximately 90% in conidiation. Microscopic examination revealed that the Δ*fgk3* mutant often produced singular phialides or conidia directly on hyphal branches or tips. In *F. graminearum*, several mutants, including the Δ*FGSG_08631*, Δ*Fgrim15*, Δ*Fgdbf2*, Δ*Fgcdc15*, Δ*FgstuA*, and Δ*FgabaA* mutants, are known to be significantly reduced in conidiation and unable to form clusters of phialides^42^,^43^. The growth rates of the Δ*Fgdbf2* and Δ*Fgcdc15* mutants were significantly reduced, while the Δ*Fgrim15* and Δ*FGSG_08631* mutants had no obvious defects in vegetative growth. Therefore, defects in growth rate are not directly related to defects in the production of conidiophores or phialides on vegetative hyphae in *F. graminearum*.

The Δ*fgk3* mutant failed to produce perithecia or protoperithecia on carrot agar plates, indicating that *FGK3* is essential for sexual reproduction in *F. graminearum*, which has ascospores as the primary inoculum. In *S. cerevisiae*, sporulation was blocked in the Δ*rim11* mutant and severely decreased in the Δ*mck1* and Δ*mrk1* mutants^44^. *RIM11* phosphorylates the master transcriptional activator Ime1, which facilitates the transcription of several early meiosis-specific genes^45^. The single GSK3 homolog in *F. graminearum* may possess similar or combined functions to its four homologs in the budding yeast and promote the transcription of numerous early meiosis-specific genes. In *S. pombe*, the *skp1* gene is not essential for meiosis or sporulation. However, in the *skp1* null mutant, the rate of sporulation was decreased, and ascospores had abnormal morphology^24^. Deletion of *FGK3* in *F. graminearum* completely blocked sexual developmental processes. Because it failed to produce protoperithecia, the Δ*fgk3* mutant must be blocked before the formation of croziers and diploid cells, which occurs prior to meiosis in Sordariomycetes.

The Δ*fgk3* mutant was significantly reduced in virulence, to the point where it was almost non-pathogenic. One contributing factor is its severe defects in hyphal growth. In addition, the Δ*fgk3* mutant was defective in DON production in inoculated wheat kernels and DON is the first and best characterized virulence factor in *F. graminearum*^26^,^27^. Furthermore, *FGK3* also plays a critical role in regulating responses to reactive oxygen species (ROS) and other environmental stresses, and it is well known that oxidative burst is a common plant defense response^46^. Increased sensitivity to environmental stresses may be another factor causing reduced virulence of the Δ*fgk3* mutant. In *C. neoformans*, disruption of *GSK3* resulted in decreased virulence in a murine intravenous model, which was mainly due to defects in the SREBP pathway^21^. Defects of the Δ*fgk3* mutant in plant infection suggest *FGK3* and its orthologs may have a conserved role in fungal pathogenesis.

Mammalian GSK3β is a multifunctional protein kinase. Its substrates are present from the cytosol to the nucleus, such as cyclin D1 in the nucleus and glycogen synthase in the cytosol^9^,^47^. Changes in the localization of GSK3β are dependent on the cell cycle, with the amount of GSK3β present in the nucleus increasing during the S phase in NIH3T3 cells. In the budding yeast, Rim11 plays a role in promoting the transcription of several meiotic genes. It is mobilized from the cytoplasm to the nucleus when cells enter the meiotic stage^48^. Our results showed that Fgk3-GFP fusion proteins had different subcellular localization patterns in conidia and hyphae, indicating that Fgk3 may also have different substrates at various growth or developmental stages in *F. graminearum*. In conidia, the fungus is in a dormant state and Fgk3 may function to inhibit glycogen synthesis by localizing to the cytoplasm. However, in growing hyphae or germ tubes, Fgk3 may localize to the nucleus to promote the transcription of various genes important for primary metabolism and cell cycle progression.

Lithium is known to inhibit the activity of mammalian GSK3β^49^. PH-1 treated with 80 mM lithium chloride had similar defects to the Δ*fgk3* mutant, except for conidiation. As a control, treatment with 80 mM KCl had no obvious effect on hyphal growth and conidium morphology in the wild type strain (Fig. 5B). Because lithium is not a specific inhibitor of GSK3 kinases, it also has other targets, including inositol monophosphatase (IMPase), phosphomonoesterase, and phosphoglucomutase^50^. The presence of LiCl may be inhibitory to these targets other than Fgk3, which may in turn reduce or eliminate the adverse effect of *Fgk3* inhibition on conidiation in *F. graminearum*. In *S. cerevisiae*, 50 mM LiCl caused defects in budding, leading to a higher amount of unbudded cells than untreated yeast cells^51^. Therefore, although lithium has a universal inhibitory effect on GSK3 kinases, its effects on growth and development may vary among different species.

The expression of *FGK3* was increased in response to cold, oxidative, and SDS stresses. In *S. cerevisiae*, *MCK1* played an important role in cell wall integrity signaling. The *mck1* mutant had increased sensitivity to environmental stresses such as elevated temperatures and 0.01% SDS^52^. However, deletion of either *MRK1* or *YGK3* had only minor effects on resistance to SDS and heat shock. General stress responses in *S*. *cerevisiae* are regulated by the transcription factor *MSN2* and its paralog *MSN4*, both of which bind to the STRE element of stress-inducible genes for various stressors^53^. We found that cold-induced up-regulation of *FgMSN2* is dependent on *FGK3* (Fig. 6D) in *F. graminearum*. In yeast two-hybrid assays, Fgk3 physically interacted with Msn2. Therefore, Fsk3 may regulate the expression of stress-response related genes, such as *FgGRE2*, *FgGPD1*, and *FgCTT1*, by directly interacting with FgMsn2 and/or affecting the expression level of FgMsn2.

Because *FGK3* is essential for plant infection and DON production, it is suitable as a candidate gene to use the host-induced gene silencing (HIGS) approach for controlling wheat head blight. Recently, silencing the *CYP51A/B/C* genes by HIGS was shown to be effective for protection against *F. graminearum* infection^54^. Therefore, it is possible that transgenic plants expressing the RNAi construct of *FGK3* will be resistant to *F. graminearum* infection or reduced in DON contamination. The Fgk3 kinase is known to interact with other protein kinases that are important for various developmental and infection processes^6^. Some of these genes functionally related to Fgk3 also can be explored as the targets for controlling Fusarium head blight.

## Methods

### Fungal strains and growth conditions

The wild-type strain of *F. graminearum* PH-1 and all other strains used in this study were routinely maintained on PDA plates cultured at 25°C. Complete medium (CM) with additions of 0.7 M NaCl, 0.05% H_2_O_2_, 0.01% SDS, or 0.3 M Congo Red were used for stress response assays. Conidiation was assayed with liquid CMC cultures as described^55^. For LiCl treatment assays, LiCl was added to PDA plates or liquid YEPD medium to final concentrations of 5, 20, 40, and 80 mM. Conidia were stained for glycogen with 60 mg/ml KI and 10 mg/ml of I_2_ as previously described^29^.

### Generation of the *FGK3*-GFP and *TUB1*-GFP fusion constructs

For complementation assays, the entire *FGK3* gene, including the promoter, was amplified by PCR with primers GSK3-GFP-F and GSK3-GFP-R (Table S2) from PH-1. The product was then cloned into *Xho*I-digested pFL2 by the yeast gap repair approach^28^. The resulting *FGK3*-GFP construct carrying the geneticin-resistant marker was isolated from yeast and transformed into the Δ*fgk3* mutant E2. G418-resistant transformants harboring the *FGK3*-GFP construct were identified by PCR and confirmed by the presence of GFP signals.

The *TUB1*-GFP construct was generated in a previous study^31^. It was transformed into protoplasts of the Δ*fgk3* mutant as described^56^. Strain T1-P10 was a *TUB1*-GFP transformant of PH-1 generated in a previous study^31^.

### qRT-PCR assays

For assaying *FGK3* expression, conidia of PH-1 were harvested from 5-day-old CMC cultures and resuspended to 10^6^ conidia/ml in liquid YEPD medium. After incubation for 16 h at 25°C, YEPD cultures were further incubated at 4°C or 37°C or in the presence of 0.01% SDS, 0.7 M NaCl, or 0.05% H_2_O_2_ for 1 h. To assay *FGK3* expression in aerial hyphae, RNA samples were isolated from PDA cultures of PH-1 that were incubated for 3 day at 25°C, then further incubated for 4 h and 20 h at 4°C or 37°C. For assaying the expression of *FgCTT1, FgGPD1, FgGRE2,* and *FgMSN2*, germlings were harvested from YEPD cultures of PH-1 and the Δ*fgk3* mutant after incubation for 16 h at 25°C and further incubated at 4°C or 37°C or in the presence of 0.7 M NaCl for 10 min and 1 h, respectively. RNA was isolated with the TRIzol reagent and used for cDNA synthesis with the Fermentas 1^st^-cDNA synthesis kit (Hanover, MD) following the instructions provided by the manufacturer. The *TUB2* gene of *F. graminearum* was amplified with primers Tub-real-F and Tub-real-R (Table S2). Changes in the relative expression level of each gene were calculated by the 2^−ΔΔCt^ method^57^ with *TUB2* as the endogenous reference. For each gene, qRT-PCR data from three biological replicates were used to calculate the mean and standard deviation.

### Sexual reproduction assays

Aerial hyphae of 7-day-old carrot agar cultures of the Δ*fgk3* mutant and PH-1 were pressed down with 0.3 M of sterile 0.1% Tween 20 as described for self-crossing^58^. Perithecium formation and cirrhi production were examined under dissect microscope after incubation at 25°C for two weeks.

### Plant infection and DON production assays

For plant infection assays, conidia from 5-day-old CMC cultures were harvested and resuspended to 10^5^/ml in sterile distilled water. Flowering wheat heads of cultivar Xiaoyan 22 were drop-inoculated with 10 μl of conidium suspensions at the fifth spikelet from the base of the spike. Symptomatic spikelets were examined and counted 14 dpi. The inoculated wheat kernels with FHB disease symptoms were harvested and assayed for DON production as described^59^. Corn silk and corn stalk infection assays were conducted as described^60^.

### Yeast two-hybrid assays

Protein-protein interactions were assayed with the Matchmaker yeast two-hybrid system (Clontech, Mountain View, CA). Whereas the *FGK3* ORF was amplified from first-strand cDNA of PH-1 and cloned into pGBK7 as the bait construct, the *FgMSN2* ORF was cloned into pGADT7 as the prey construct. The resulting bait and prey vectors were co-transformed into the yeast strain AH109. The Leu^+^ and Trp^+^ transformants were isolated and assayed for growth on SD-Trp-Leu-His medium and galactosidase activities in filter lift assays. The positive and negative controls were provided in the Matchmaker Library Construction & Screening Kits (Clontech).

## Author Contributions

J.Q., G.W. and C.J. performed the experiments, participated in the analysis of the data. J.X. was involved in experimental designs, data interpretation, and manuscript preparation. C.W. designed the experiments, participated in data analysis, and wrote the manuscript. All authors read, corrected and approved the final manuscript.

## Supplementary Material

Supplementary Information Supplemental Figures S1-S5 and Tables S1-S2

## Figures and Tables

**Table 1:** Disease index and DON production in the wild type and mutant strain

Strain	Disease Index a	DON (ppm) b
PH-1 (wild type)	15.27 ± 2.05^A ^*	1629.30 ± 161.27^A^
E2 (Δ*fgk3*)	0.18 ± 0.40^B^	nd
C1 (Δ*fgk3/FGK3*-GFP)	15.91 ± 8.32^A^	1653.00 ± 164.05^A^

^a^Disease index was calculated from the number of symptomatic spikelets per wheat head examined 14 dpi. At least 10 wheat heads were examined in each replicate.

^b^DON production was measured with the inoculated wheat kernels that developed FHB symptoms 14 dpi.

*Mean and standard deviations were calculated with results from three independent experiments. Data were analyzed with SPSS One-way ANOVA analysis. Differing letters denote a statistically significant difference (P = 0.05).

nd, not detectable.
