# Supplementary material for: Fgk3 glycogen synthase kinase is important for development, pathogenesis, and stress responses in Fusarium graminearum
Source: Sci Rep. 2015 Feb 23;5:8504. doi: 10.1038/srep08504 (PMC4336942; doi:10.1038/srep08504)
Supplement: Supplementary Information — Supplemental Figures S1-S5 and Tables S1-S2 [file srep08504-s1.pdf]

Fgk3 glycogen synthase kinase is important for development, pathogenesis, and stress responses in *Fusarium graminearum*

Jun Qin[1†], Guanghui Wang[1†],Cong Jiang[1], Jin-Rong Xu[2], and Chenfang Wang[1*]

[1] State Key Laboratory of Crop Stress Biology for Arid Areas, College of Plant Protection, Northwest A&F University, Yangling, Shaanxi, China.

[2]Department of Botany and Plant Pathology, Purdue University, USA.

[†] These two authors contributed equally.

*Corresponding authors: Chenfang Wang
Tel: 86-029-8708-1270
Email: wangchenfang@nwsuaf.edu.cn

Figure S1

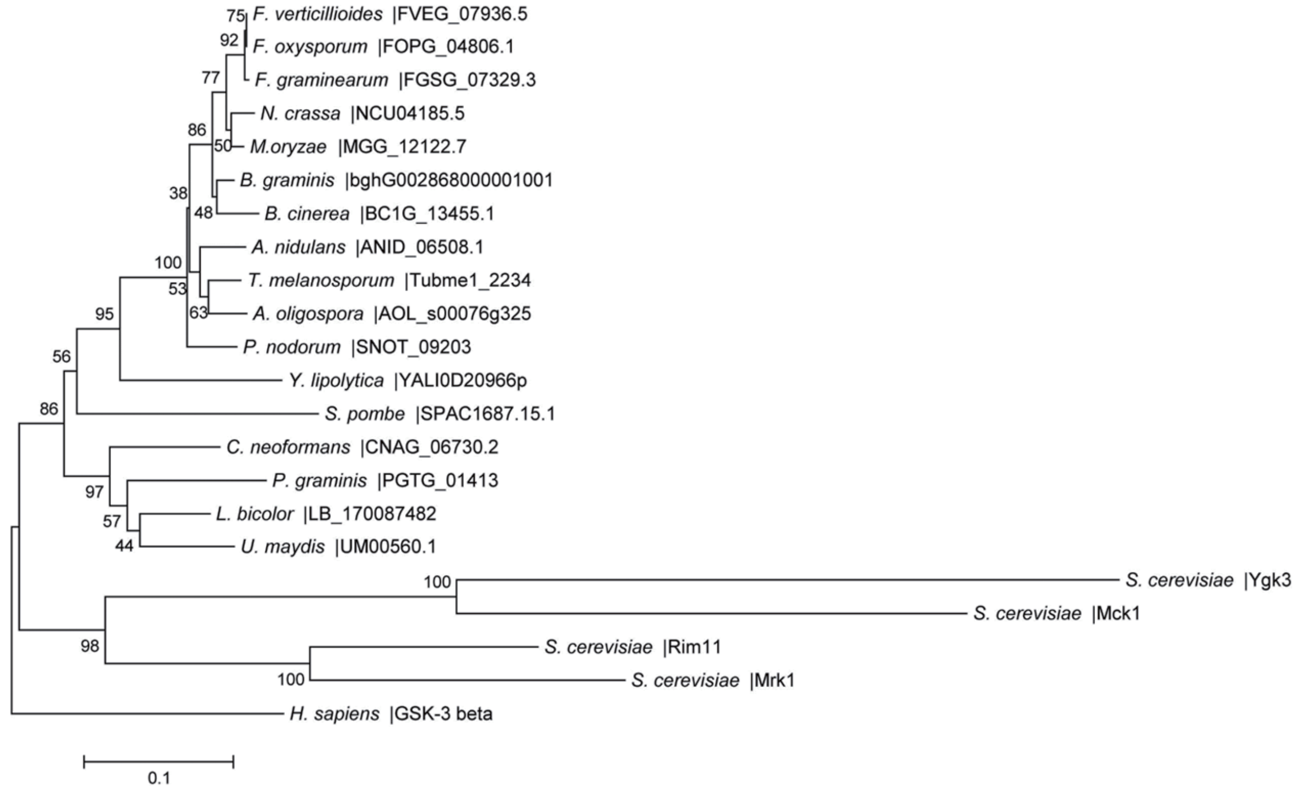

Figure S1. Phylogeny of representative fungal GSK3 kinases. *Fusarium verticillioides*, *Fusarium oxysporum*, *Fusarium graminearum*, *Neurospora crassa*, *Magnaporthe oryzae*, *Blumeria graminis*, *Botrytis cinerea*, *Aspergillus nidulans*, *Tuber melanosporum*, *Arthrobotrys oligospora*, *Phaeosphaeria nodorum*, *Yarrowia lipolytica*, *Schizosaccharomyces pombe*, *Cryptococcus neoformans*, *Puccinia graminis*, *Laccaria bicolor*, *Ustilago maydis*, *Saccharomyces cerevisiae*, and *Homo sapiens*. Human GSK3 is included as the out-group.

Figure S2

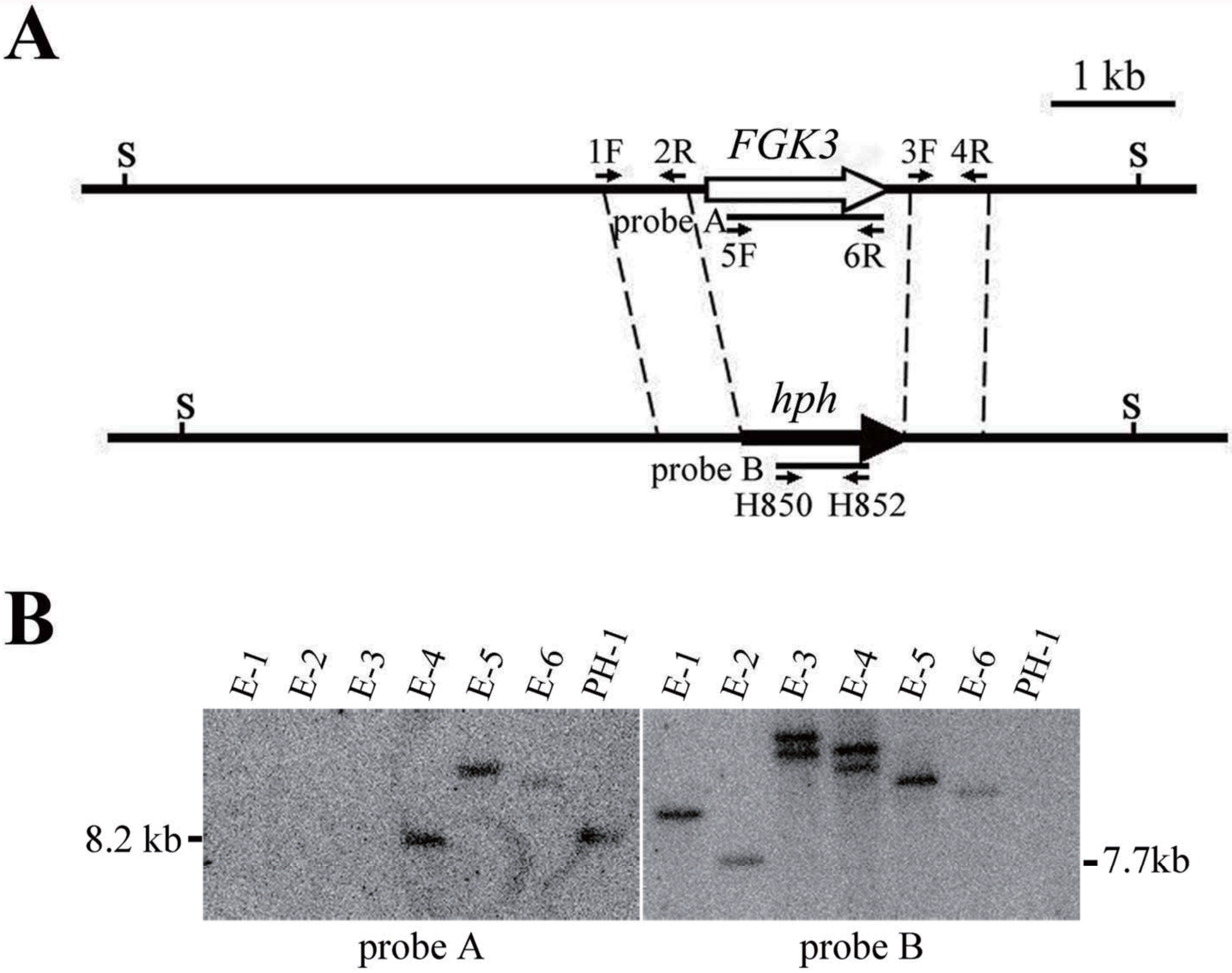

Figure S2. Construction of *FGK3* gene replacement and the Δ*fgk3* mutant. A. The *FGK3* locus and gene replacement construct. The empty and black arrows refer to the *FGK3* gene and the *hph* gene, respectively. Primers 1F/2R and 3F/4R were used to amplify the flanking sequences of *FGK3*. Probes A and B were amplified with 5F/6R and H850/H852, respectively. S, *Sal*I. **B.** Southern blot analysis of the wild type (PH-1) and Δ*fgk3* transformants (E-1 to E-6). All DNA samples were digested with *Sal*I. The blots were hybridized with probe A (left), amplified with primers 5F/6R, and probe B (right), amplified with H852/H850.

Figure S3

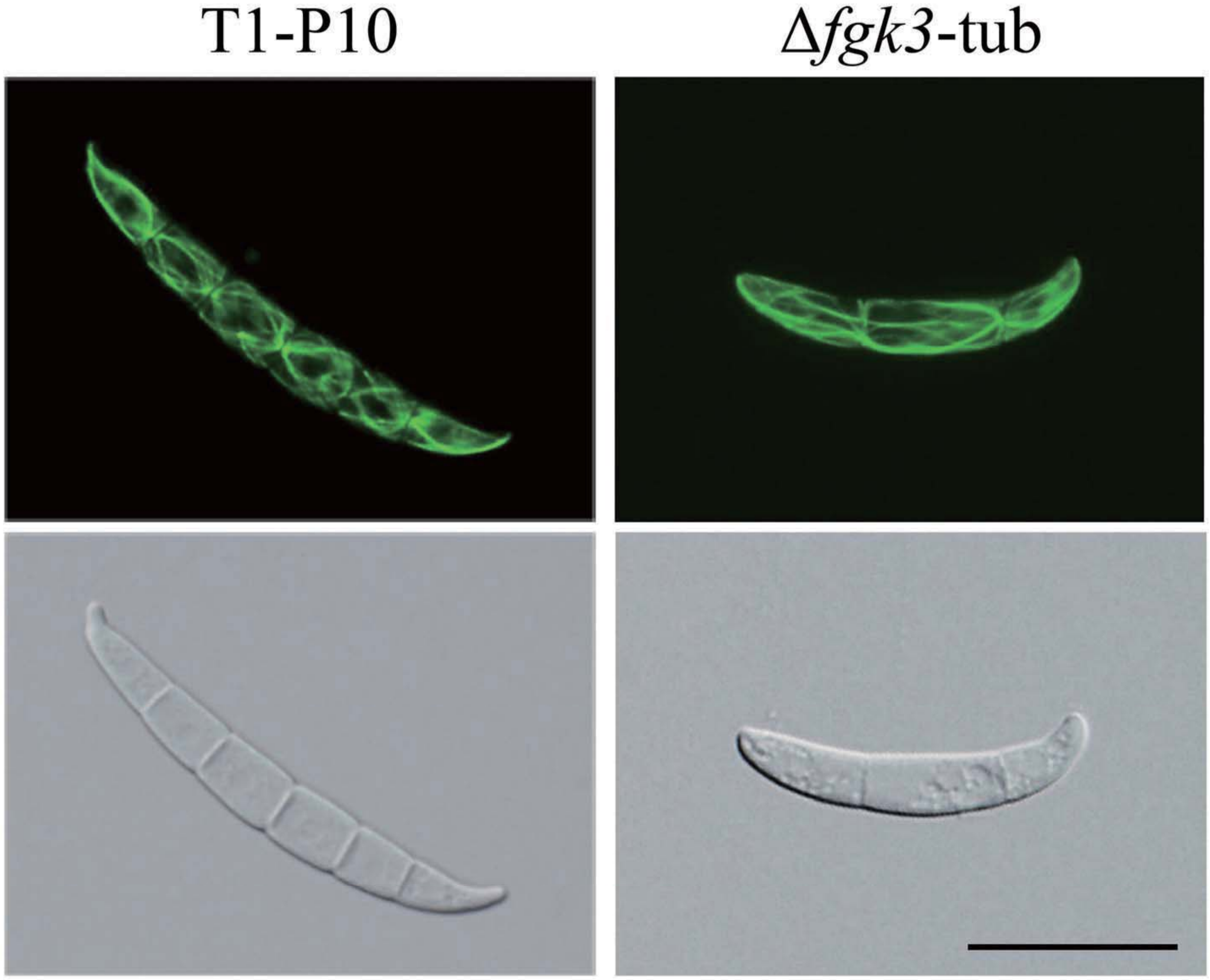

Figure S3. Defects of microtubule bundling at the septal pore in the Δ*fgk3* mutant. Conidia of the transformants of PH-1 (T1-P10) and the Δ*fgk3* mutant (Δ*fgk3*-tub) expressing the TUB1-GFP construct. Both strains were examined by epifluorescence and DIC microscopy.

Figure S4

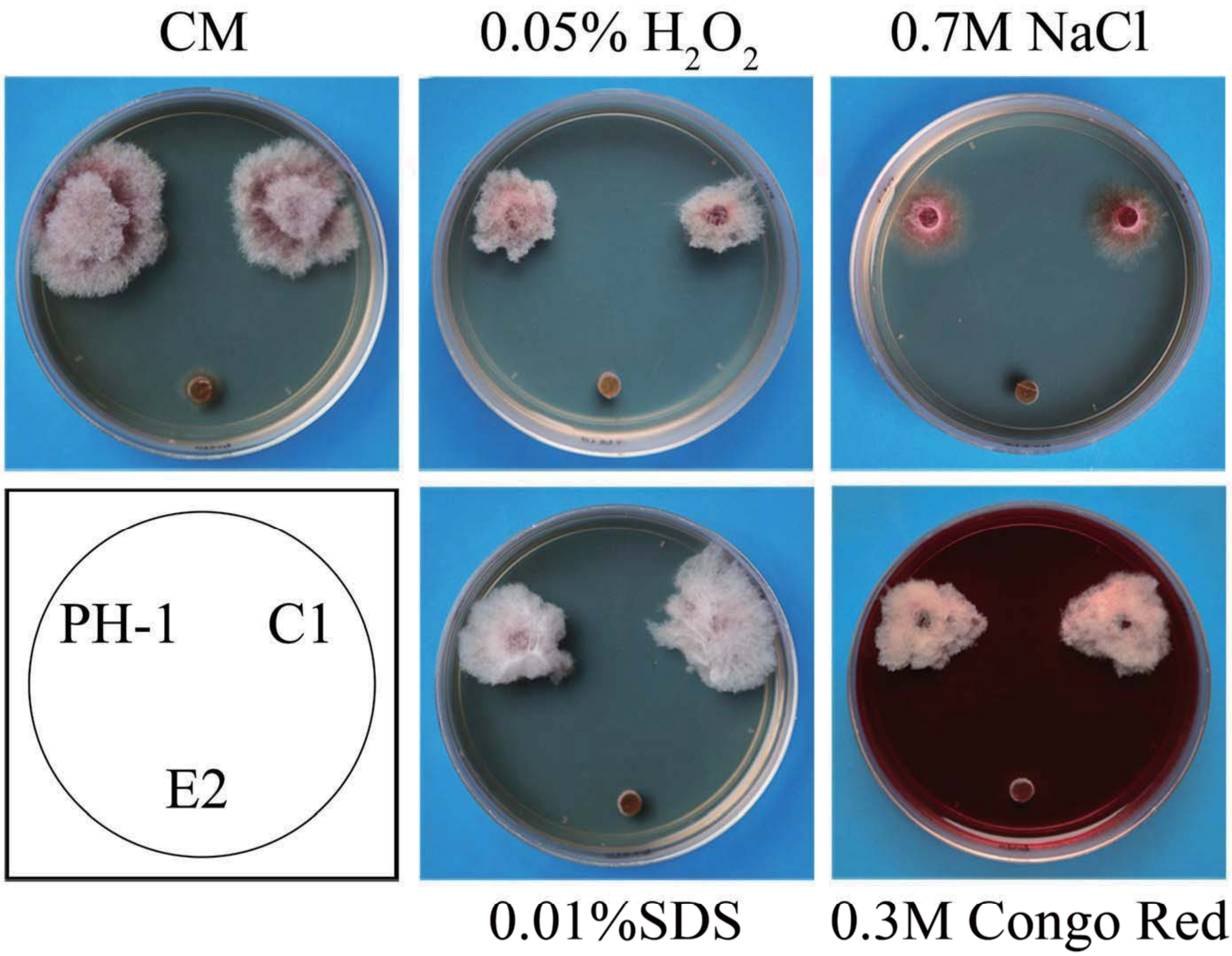

Figure S4. Assays for growth on PDA in the presence of different stresses. PH-1, Δ*fgk3* mutant (E2), and complementation strain (C1) were cultured on CM plates with 0.7 M NaCl, 0.05% H_2O_2, 0.01% SDS, and 0.3 M Congo Red. Photographs were taken 3 days after incubation at 25℃.

Figure S5

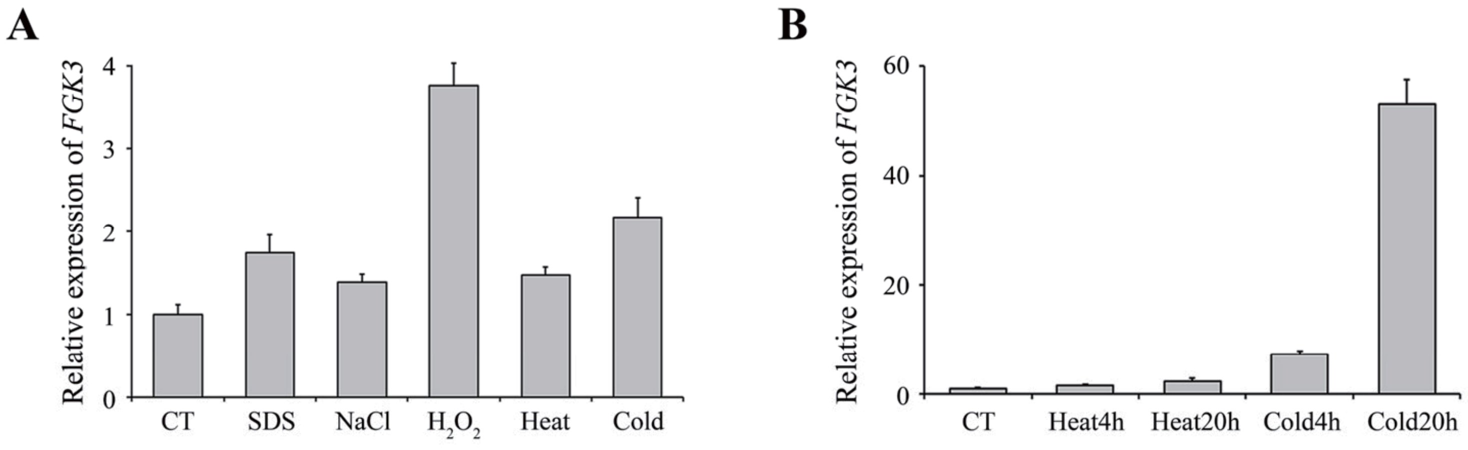

Figure S5. Expression of *FGK3* in response to different stresses in PH-1. A. Expression of *FGK3* in liquid cultures grown under different environmental conditions. RNA samples were isolated from germlings of PH-1 incubated in YEPD cultures for 16 h at 25°C further incubated at 4°C or 37°C or in the presence of 0.01% SDS, 0.7 M NaCl, or 0.05% H_2O_2 for 1 h. The expression level of *FGK3* in regular YEPD cultures incubated at 25°C was arbitrarily set to 1. **B.** Expression of *FGK3* in aerial hyphae in response to cold or heat shock. RNA was isolated from PDA cultures that were incubated at 25°C for three days and then further incubated for 4 h and 20 h at 4°C or 37°C. The expression level of *GFK3* in normal PDA cultures grown at 25°C was arbitrarily set to 1. CT: control, untreated PH-1 cultures. Mean and standard deviation were calculated with results from three independent replicates.

Table S1. Effects of 80 mM LiCl on growth rate and conidiation.

Strain	Growth rate (mm/d)[a]	Conidiation ($\times 10^5$ conidia/ml)
PH-1 (untreated)	11.56±0.10[A*]	9.00±2.80[A]
PH-1 (LiCl treated)	0.61±0.10[C]	8.96±2.49[A]
E2 (Δ*fgk3*)	1.33±0.34[B]	0.91±0.25[B]

[a] Growth rate and conidiation were measured with 3-day-old CM cultures and 5-day-old CMC liquid cultures, respectively.

[*] Mean and standard deviations were calculated with results from three independent experiments. Data were analyzed with SPSS One-Way ANOVA analysis. Different letters denote a statistically significant difference (P = 0.05).

Table S2. PCR primers used in this study

Name	Sequence (5'-3')
1F	cactctctttctgccttgcttccat
2R	ttgacctccactagctccagccaagccttggatagcgatgctccttcaatgg
3F	gaatagagtagatgccgaccgcgggttccagaccaagaccggaccagat
4R	tgtcagttacgaaacggcaacgatt
5F	gctctttcggtgtcgttttccag
6R	cagtttcgccatcatctcctgct
7F	ctgcgtcttctctggctg
8R	cctgagcgttgtgtttgcc
YG-F	gatgtaggagggcgtggatatgtcct
HY-R	gtattgaccgattccttgcggtccgaa
HYG-F	ggcttggctggagctagtggaggtcaa
HYG-R	aacccgcggtcggcatctactctattc
H852	aactcaccgcgacgtctgtc
H850	ttgtccgtcaggacattgtt
H856F	gtcgatgcgacgcaatcgt
H855R	gctgatctgaccagttgc
GSK3-CM-F	ctggcatccaaacgtcgatagcacg
GSK3-CM-R	cctttgccgcagcattgggttc
GSK3-GFP-F	cgactcactatagggcgaattgggtactcaaattggggcatccaaacgtcgatagcacg
GSK3-GFP-R	caccaccccggtgaacagctcctcgcccttgctcacatccagtttcgccatcatctcct
Tub1-eGFP-F	cgactcactatagggcgaattgggtactcaaattgggatgggtgtggtatttacgg
Tub1-eGFP-R	caccaccccggtgaacagctcctcgcccttgctcacctcctcgccctcaggcag
GSK3-real-F	ggatggaattaccggcgaga
GSK3-real-R	gcagcatcctctcctgatgg
Tub-real-F	ggtcagtgcggtaaccaaatcg
Tub-real-R	ggagctcagaggtgccgttgtaa
GPD1-real-F	agtacctaccgggcattcct
GPD1-real-R	taaactggtggggcaggttg
GRE2-real-F	acgaaagatagcgggcaagt
GRE2-real-R	agctgtgacagtaatccagacat
CTT1-real-F	cagtcggaggagagtctggat
CTT1-real-R	gttagataaccctcctcagttcg
